# Influence of the Sintering Method on the Properties of a Multiferroic Ceramic Composite Based on PZT-Type Ferroelectric Material and Ni-Zn Ferrite

**DOI:** 10.3390/ma15238461

**Published:** 2022-11-28

**Authors:** Dariusz Bochenek, Artur Chrobak, Grzegorz Dercz

**Affiliations:** 1 Institute of Materials Engineering, Faculty of Science and Technology, University of Silesia in Katowice, 75 Pułku Piechoty 1a, 41-500 Chorzów, Poland; 2 Institute of Physics, Faculty of Science and Technology, University of Silesia in Katowice, 75 Pułku Piechoty 1a, 41-500 Chorzów, Poland

**Keywords:** multiferroics, ferroelectric-ferromagnetic composites, perovskite-type materials, dielectric properties, magnetic properties

## Abstract

This paper presents the research results of multiferroic ceramic composites obtained with three sintering methods, i.e., free sintering FS (pressureless), hot pressing HP, and spark plasma sintering SPS. The multiferroic composite was obtained by combining a ferroelectric material of the PZT-type (90%) and zinc-nickel ferrite (10%). Research has shown that the combination of a magnetic material and ferroelectric materials maintains the multiferroic good ferroelectric and magnetic properties of the composites for all sintering methods. A sample sintered with the HP hot pressing method exhibits the best parameters. In the HP method, the composite sample has high permittivity, equal to 910 (at room temperature) and 7850 (at the phase transition temperature), residual polarization 2.80 µC/cm^2^, a coercive field of 0.95 kV/mm, and the magnetization of 5.3 and 4.95 Am^2^/kg at −268 °C and *RT*, respectively. Optimal technological process conditions are ensured by the HP method, improving the sinterability of the ceramic sinter which obtains high density and proper material compaction. In the case of the SPS method, the sintering conditions do not allow for homogeneous growth of the ferroelectric and magnetic component grains, increasing the formation of internal pores. On the other hand, in the FS method, high temperatures favor excessive grain growth and an increase in the heterogeneity of their size. In obtaining optimal performance parameters of multiferroic composites and maintaining their stability, hot pressing is the most effective of the presented sintering methods.

## 1. Introduction

Multiferroics and multiferroic ceramic composites are an increasingly developing field of materials engineering (the area of materials with functional properties) for wide applications in microelectronics and micromechatronics [1,2,3]. However, ever greater requirements and newer applications of materials with functional properties in modern materials engineering make it necessary to obtain ceramic materials with reliable and stable parameters. In addition to searching for new types of multiferroic materials, an intensive search for effective technological methods for their production (synthesis and sintering) to meet these unique requirements has been underway [1,4]. Numerous synthesis methods of ceramic materials are known, e.g., solid-state reaction technique [5,6,7,8], calcination [9,10,11,12,13], sol-gel [14,15], gel-combustion [16], mechanochemical activation [17,18,19,20], and the self-developing synthesis of SHS [21,22], as well as sintering methods, e.g., free sintering (pressureless sintering) [23], hot pressing [24,25,26], microwave sintering [23,27,28], spark plasma sintering SPS [29,30,31,32], and cold-sintering-assisted sintering CSS [33,34]. Well-known methods of synthesis and sintering are used to improve the functional properties of multiferroic materials, which are appropriately modified and combined in the technological process. It is widely known that, during the technological process, the selection of the method and technological conditions (for both synthesis and sintering) affects the microstructure and crystal structure of ceramic materials [35,36]. On the other hand, the crystal structure and microstructure of ceramic materials have a decisive influence on their physical properties, as well as the temperature and time stability of their valuable parameters [36]. One way to obtain functional materials with multiferroic properties is to combine a material with high ferroelectric properties and a material with magnetic properties, in order to form a ceramic composite [37,38,39,40,41,42,43]. Increased application possibilities of this type of material depend on the coupling of the magnetic and electrical subsystems [1,2,4,44,45,46,47,48]. Previous investigations have shown that the magnetoelectric coupling coefficient in multiferroic ferrite composites is higher for compositions with higher ferrite content in the composite material [48,49]. However, the presence of ferrite causes a significant deterioration of the ferroelectric properties, which makes it necessary to design a type of multiferroic material with a predominance of the ferroelectric phase [50].

The best-known and widely used material with ferroelectric and piezoelectric properties is the solid solution Pb(Zr_1-*x*_Ti*_x_*)O_3_ (PZT) which is most often obtained with a solid-state reaction technique [5,6]. The properties of this material mainly depend on the Zr/Ti content ratio. The improvement of the physical properties of the PZT-type materials can be achieved, inter alia, by doping the base composition with appropriate admixtures [9,51,52,53,54,55], designing multi-component solid solutions [52,56,57], as well as using unique technological methods [58,59,60,61,62]. PZT-type materials with both tetragonal and rhombohedral phases exhibit extremely high (or low) values of physical properties, which is particularly interesting in terms of their application [63,64]. In this work, a doped solid solution of the PZT-type was used to obtain multiferroic composite materials.

In the case of magnetic materials, ferrites with different properties (obtained on the basis of nickel, zinc, manganese, or cobalt) can be good components of multiferroic composites. Due to the relatively high resistivity of the magnetic materials, nickel-zinc ferrites seem to be one of the most suitable for the abovementioned applications. The nickel-zinc ferrite Ni_0_._64_Zn_0_._36_Fe_2_O_4_ belongs to a group of soft ferrites with high values of the magnetic properties *B* = 380 mT, *μ_i_* = 125 (at 20 °C), high resistivity (~10^5^ Ωm at 25 °C), and a working frequency range of 50–1000 MHz, high Curie temperature, and chemical stability [65,66]. This type of ferrite is used for low and higher-frequency applications; e.g., as power transformers and inductors, microwave devices, telecom filters, delay lines, EMI-suppression, wide-band transformers, etc. [67]. In the technological process, several sintering methods are used to obtain multiferroic composite materials, including free sintering (pressureless sintering), hot pressing, and spark plasma sintering [32,35,39,40,68,69]. Each of these sintering methods has both numerous advantages as well as disadvantages, whereas not all types of materials subjected to sintering can achieve the desired effect and optimal final properties.

The current study aimed at obtaining multiferroic composites based on a PZT-type ferroelectric powder and a ferrite powder, using three sintering methods, as well as studying the effect of sintering on their physical properties. The research presented in the paper was intended to select a sintering method characterized by the high sinterability of composite powders, which would allow for obtaining an appropriate microstructure of the ceramic materials. The correct microstructure is conducive to maintaining sufficiently high electrophysical parameters (including dielectric properties) and resistivity of the multiferroic composites, minimizing their deterioration due to magnetic addition in the composition of the composite. In this study, the percentage of multiferroic composites was 90/10 (ferroelectric/paraelectric).

## 2. Experimental

### 2.1. Research Material 

The work presents research results of the multiferroic composite samples obtained with three different sintering methods, i.e., spark plasma sintering (SPS), hot pressing (HP), and free sintering (FS), all of which were compared to the PZT material (constituting the matrix of the composite material). The PZT-type ceramic material (Pb_0_._90_Ba_0_._10_(Zr_0_._53_Ti_0_._47_)O_3_+ 2%at.Nb_2_O_5_) was obtained using classic technology (solid state reaction technique), using a mixture of simple oxides PbO (99.99%, POCH, Gliwice, Poland), BaCO_3_ (99.99%, POCH, Gliwice, Poland), Nb_2_O_5_ (99.9%, Sigma-Aldrich, St. Louis, MO, USA), ZrO_2_ (99.5%, Aldrich, St. Louis, MO, USA), and TiO_2_ (99.99%, Merck, Darmstadt, Germany). The powders were mixed in a planetary ball mill, Fritsch Pulverisette 6 (Idar-Oberstein, Germany), for 15 h (wet method). The synthesis was carried out under the following conditions: 950 °C/8 h. The ceramic sample was obtained by the free sintering (pressureless) method under conditions: 1250 °C/2 h. Silver electrodes were applied to both surfaces of the ceramic sample, in order to carry out electric tests.

The ferrite Ni_0_._64_Zn_0_._36_Fe_2_O_4_ material was obtained from simple oxides: NiO (99.99%, Aldrich, Steinheim, Germany), Fe_2_O_3_ (99.98%, Sigma-Aldrich, St. Louis, MO, USA), and ZnO (99.99%, Aldrich, Steinheim, Germany), which were mixed in a planetary ball mill (Fritsch Pulverisette 6) for 12 h. Subsequently, the mixture of powders was synthesized at 1100 °C/4 h (calcination route), and the nickel-zinc ferrite powder was obtained. 

Multiferroic composites were prepared by combining the synthesized PZT powder with the ferrite powder, in the proportion of 90:10 (P/F) using three sintering methods. After weighing, the powders were mixed in a planetary ball mill, Fritsch Pulverisette 6 (Idar-Oberstein, Germany), for 24 h (wet method). Subsequently, the multiferroic powder was synthesized under the following conditions: 1050 °C/4 h. 

#### 2.1.1. Spark Plasma Sintering Method

The synthesized PF composite powders were sintered by the spark plasma sintering (SPS) method using an SPS machine manufactured by FCT System GmbH, model HP D5 [68]. The SPS process conditions were as follows: temperature 900 °C, dwell time 3 min, pressure 50 MPa, atmosphere argon gas, heating rate 50 °C/min, and pressing force 4 kN applied uniformly to the die punch during the SPS process. During the SPS process, a number of its technological parameters were recorded, i.e., temperature, time, heating and cooling rates, pressure force, etc., and the changes in the resistance of the sintered material were visualized [32]. These parameters allowed for selecting optimal parameters for a specific material. The selection of technological conditions for the SPS process was made based on experimental research presented in previous studies [32,69]. A more detailed description of the SPS method is presented in [68]. The multiferroic composite sample sintered by the SPS method was labeled as PF-SPS.

#### 2.1.2. Hot Pressing Method

The synthesized PF composite powders were sintered by the hot pressing method (HP). In the HP process, the multiferroic powder was pressed into a plate mold and consecutively placed in a die in the furnace chamber using a protective powder. The final densification was carried out under conditions: 1200 °C/1 h/20 MPa. The device uniformly and simultaneously applied pressure with increasing temperature. The selection of technological conditions of the HP process was made based on experimental research presented in [70,71]. The multiferroic composite sample sintered by the HP method was labeled as PF-HP.

#### 2.1.3. Free Sintering Method

The synthesized PF composite powders were sintered using the free sintering method (FS), i.e., pressureless sintering (a method commonly used for sintering ceramic materials). In the FS process, the multiferroic powder was pressed into a plate mold and placed in a ceramic crucible surrounded by protective powder. Pressureless sintering was performed in a furnace under 1250 °C/2 h. The multiferroic composite sample sintered by the FS method was labeled as PF-FS. The selection of technological conditions for the FS process was made based on experimental research presented in [70,71,72].

After the technological process, the ceramic samples were ground and polished, and then silver electrodes were placed on their measuring surfaces for electrical tests. 

### 2.2. Investigations

The XRD measurement of the composite sample was performed at room temperature (*RT*) using an X’Pert Pro diffractometer (PANalytical, Eindhoven, Netherlands) with CuK*_α_*= 1.54056 Å radiations, at the range of 2*θ*, from 14° to 66°, in the step-scan mode: 0.05 degrees and 4 s/step, and the copper radiations CuK*_α_*. Phase identification was made according to the ICDD PDF-4 (International Center for Diffraction Data Powder Diffraction Files) database. The morphology of the surfaces of the ceramic materials were analyzed by scanning electron microscopy, JSM-7100F TTL LV (Jeol Ltd., Tokyo, Japan). Two image capture techniques were used, i.e., the BSE technique (signals from the backscattered electron detectors) and the SB standard method (both signals from the secondary and backscattered electron detectors). Point, linear, and surface analyses of the chemical composition were performed using energy dispersive spectrometry (EDS, Jeol Ltd., Tokyo, Japan). The distribution of elements on the surface of the composite samples were determined by electron probe microanalysis (EPMA)—Jeol Ltd., Tokyo, Japan). For microstructure analysis, the surfaces of the samples were covered with a gold layer (Smart Coater DII-29030SCTR, Jeol Ltd., Tokyo, Japan). The average grain size was designated using the ImageJ program. The relative density of the ceramic samples was specified according to the Archimedes method. The dielectric properties were carried out with a QuadTech 1920 Precision LCR Meter (Maynard, MA, USA), at a temperature range from *RT* to 450 °C and a frequency range from 20 Hz to 1 MHz (a heating rate of 2 deg./min). The DC electrical conductivity was performed with a Keithley 6517B electrometer (Cleveland, OH, USA) in a temperature range from *RT* to 420 °C. Ferroelectric tests (*P-E* hysteresis loop) were carried out with a Sawyer-Tower circuit (using an A/D, D/A transducer card—National Instruments Corporation) and a high voltage amplifier (Matsusada Inc. HEOPS-5B6 Precision (Kusatsu, Japan). The magnetic properties of the composite samples were conducted in the low-temperature range (from −268 °C to 130 °C) using a SQUID (MPMS XL-7 Quantum Design, San Diego, CA, USA) magnetometer in a range of external magnetic field ±7 T. 

## 3. Results and Discussion

### 3.1. Properties of the PZT-Type Material

**Figure 1:**
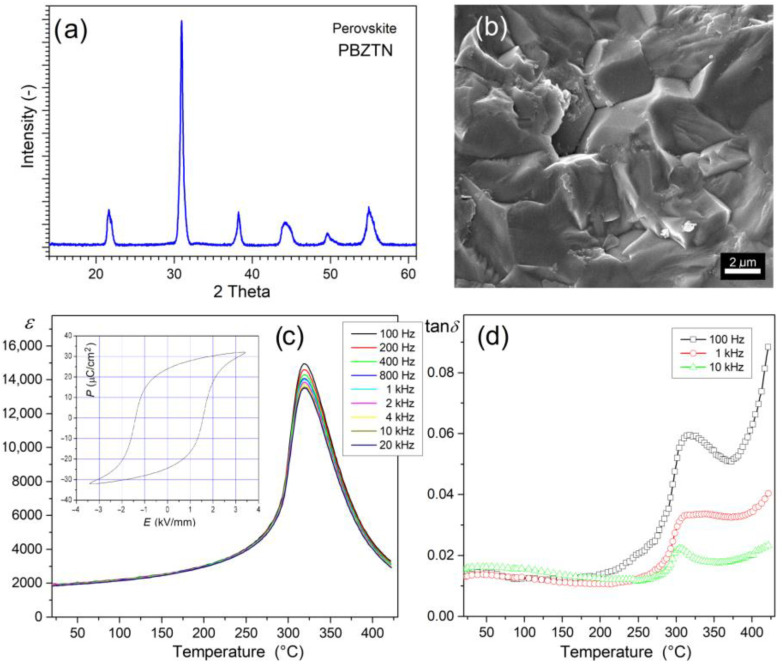
X-ray diffraction (**a**), SEM microstructure (**b**), temperature dependence of permittivity (**c**), and dielectric loss factor (tan*δ*) (**d**) for P ceramics. Inside (Figure 1c) ferroelectric hysteresis loop.

Figure 1 presents the X-ray diffraction and SEM tests, as well as dielectric and ferroelectric properties of the ferroelectric material Pb_0_._90_Ba_0_._10_(Zr_0_._53_Ti_0_._47_)O_3_ + 2%at.Nb_2_O_5_ (P), i.e., the matrix element of composite materials. The material exhibits a perovskite-type structure both from tetragonal and rhombohedral phases (i.e., a morphotropic area closer to the rhombohedral phase). The coexistence of phases is indicated based on broad reflex (before 2*θ* = 45°) with the rhombohedral (ICDD 01-073-2022) and tetragonal peaks (ICDD 00-033-0784). The microstructure of the ferroelectric P ceramics is characterized by a firmly compacted structure and tightly packed grain. The sample breaks through the grain, creating a firmly solidified and uniform grain structure with grain boundaries that are not clearly visible. In some places on the ceramic sample, the fracture reveals individual grains on the surface morphology (Figure 1b).

Ferroelectric P material has excellent dielectric properties and high resistivity at *RT* (2.20 × 10^10^ Ωm). High values of permittivity occur both at *RT* (*ε* = 1910, for 1 kHz) and at the phase transition temperature (*ε_m_* = 14,020, for 1 kHz). The phase transition (from the ferroelectric phase to the paraelectric phase) takes place in a narrow temperature range (*T_m_* = 592 °C). At the same time, the dielectric loss expressed as the tangent of the dielectric loss angle (dielectric loss factor) remains at low values, even up to 400 °C (Figure 1c). The P material also exhibits high piezoelectric and ferroelectric parameters. The *P-E* hysteresis loop is wide, characteristic of perovskite ferroelectrically hard materials (with the coercive field *E_c_* = 1.54 kV/mm), with high values of spontaneous polarization (*P_s_* = 32 μC/cm^2^) and residual polarization (*P_r_* = 27.5 μC/cm^2^). Additionally, the doped PZT-type materials show a high temperature and time stability in terms of their electrophysical parameters [73]. The excellent physical properties of this ceramic material predispose it to design multiferroic ceramic composites as a ferroelectric matrix component.

### 3.2. Structure Tests of Composite Samples

**Figure 2:**
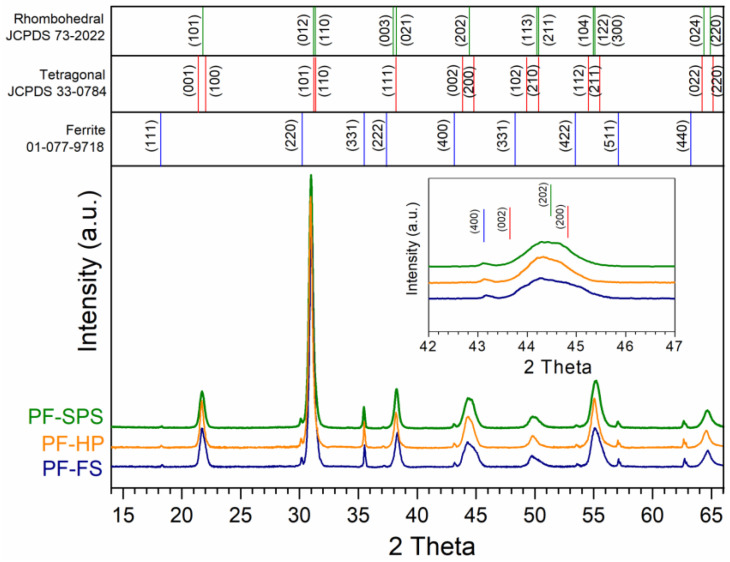
X-ray diffraction patterns for the PF multiferroic composites.

Figure 2 depicts the X-ray diffraction patterns for the PF composite samples measured at *RT*. Two sets of well-defined peaks, corresponding to the ferroelectric (P) and magnetic (F) phases, were clearly identified for all tested PF composite samples. In the case of the P ferroelectric component, the XRD analysis showed the coexistence of two phases, i.e., the tetragonal phase (with good pattern matching to ICDD 00-033-0784) and the rhombohedral phase (with good pattern matching to ICDD 01-073-2022), closer to the rhombohedral phase. The identified phases have the following space groups: P*4mm* and R*3m*, respectively [73]. The peak occurring before 2*θ* = 45°, is not sharp but blurred (Figure 2), which confirms the presence of two phases in the structure of the material. In the case of the ferrite material (Ni_0_._64_Zn_0_._36_Fe_2_O_4_), the X-ray diffraction patterns show a cubic spinel crystal structure (space group F*d*−*3m*) with good pattern matching ICDD 01-077-9718.

### 3.3. Microstructure Measurements of Composite Samples 

**Figure 3:**
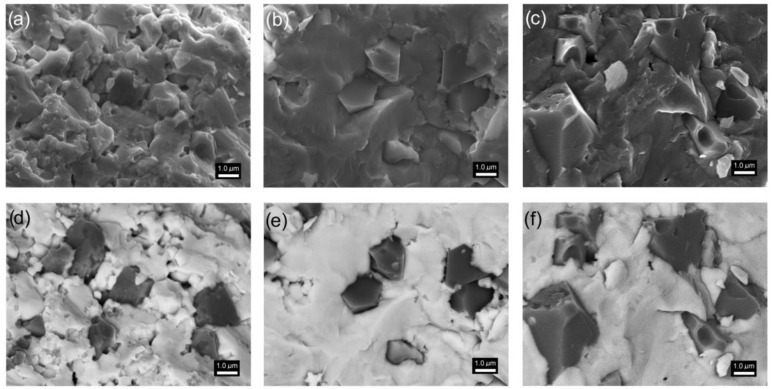
SEM images of the PF multiferroic composites: (**a**,**d**) PF-SPS, (**b**,**e**) PF-HP, and (**c**,**f**) PF-FS, made using the standard SB method (**a**–**c**) and BSE method (**d**–**f**).

Multiferroic composites obtained based on the ferroelectric P material (composite matrix) and ferrite have a similar microstructure appearance to P ceramics. In the morphology of the composite surface, it is possible to distinguish exposed grains with regular and distinct boundaries (Figure 3a–c). The correct separation of magnetic grains and ferroelectric matrix grains, as well as complete visualization of the distribution of magnetic grains in the composite matrix, is possible due to the BSE image capture technique (signals from the backscattered electron detectors). In this method, zones with a group of elements with a lower mass are depicted as dark areas (ferrite grains). In contrast, zones with a group of higher elements are depicted as bright regions (ferroelectric grains). Observation of the backscattered electrons allows the differences in the composition of the multiferroic composite sample to be visualized using different levels of contrast (Figure 3d–f). The SEM BSE image was captured from the same area of the sample surface as in the case of the SEM SB standard technique.

**Figure 4:**
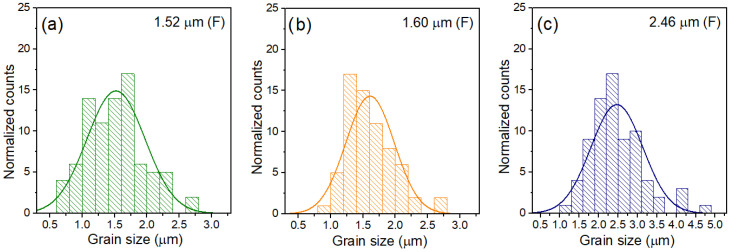
Grain size distribution of the magnetic grains in the ferroelectric composite matrix for the PF composites: PF-SPS (**a**), PF-HP (**b**), and PF-FS (**c**).

The microstructure of the composite sample obtained with the SPS method (PF-SPS) is fine-grained (Figure 3a,d). The matrix grains (ferroelectric phase) are strongly agglomerated, and their breakage occurs through the grain. Fine grains predominate; however, there are also much larger grains, which makes the microstructure heterogeneous in terms of grain size. The sintering conditions used in the SPS method (suitable for other multiferroic composite materials of this type, e.g., [32,69]) do not effectively affect the uniform grain growth for use in the present ferroelectric P component, which results in the formation of internal pores in the sample volume. It decreases the composite sample density. In contrast, the grains of the magnetic component are much larger with clearly visible grain edges. It shows that the sintering conditions used in the SPS process for the magnetic component are correct. The average size of the magnetic grains for the PF-SPS sample is 1.52 µm (Figure 4, Table 1). 

In the case of the composite sample (PF-FS) obtained by the FS method, in the process in which the highest sintering temperatures were applied, the magnetic grains are enormously expanded and show significant grain size heterogeneity (Figure 3c,f). The magnetic grains of the ferrite component with a characteristic pyramidal shape grow into the composite phase matrix and are randomly distributed (Figure 3f). The grain boundaries have clearly visible edges. The average size of the magnetic grains for the PF-FS sample is 2.46 µm (Figure 4). In contrast, the ferroelectric grains constitute a strongly sintered microstructure creating a firmly solidified surface.

The most ordered composite microstructure is for the multiferroic composite sample obtained by hot pressing method (PF-HP)—Figure 3b,e. Adequately reducing the sintering temperature and sintering time during the technological process (compared with the FS method), with the simultaneous application of external pressure, has a positive effect on both the ferroelectric and magnetic components of the multiferroic composite materials. In the case of the ferroelectric matrix, the surface morphology is similar to that of the P ceramics with tightly compacted and solidified grains without visible boundaries. On the other hand, the magnetic grains do not grow in an uncontrolled manner, as occurs during the FS method. However, they show greater grain size uniformity in the volume of the composite microstructure (Figure 3e). In addition, the edges of the magnetic grains are clearly visible, and the grains are of a regular shape. The average size *r_a_* of the magnetic grains for the PF-HP sample is 1.60 µm (Figure 4).

**Figure 5:**
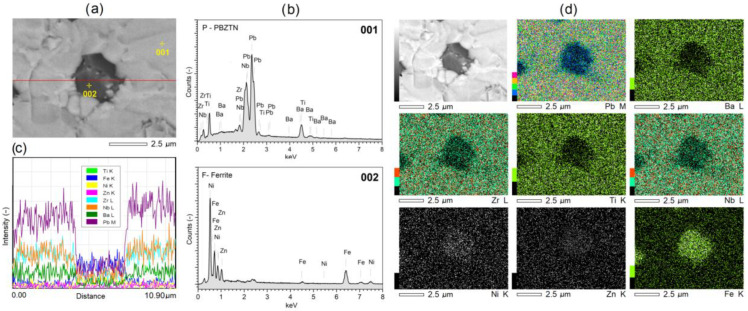
SEM/BSE microstructure image (**a**) subjected to (**b**) point and (**c**) linear EDS analysis and (**d**) EPMA maps of the distribution of individual elements in the PF-HP composite sample.

Figure 5 depicts a set of microstructure analyses of the exemplary PF-HP composite sample, i.e., SEM images captured by the BSE technique, lines and point EDS analyses, and EPMA tests. The point EDS analysis (Figure 5a,b) showed that the bright areas visible in the SEM BSE images belong to the ferroelectric phase (001—Figure 5b), i.e., elements with a higher atomic number, while the dark ones belong to the magnetic phase (002—Figure 5b), i.e., elements with a lower atomic number. The linear EDS analysis (Figure 5a,c) registers a change in intensity of the waveforms for individual elements in the variable measurement region (according to the red line in Figure 5a). In the area of the ferrite grain, there is a decrease in the intensity of the signal coming from the ferroelectric phase (minima of the Pb, Ba, Zr, Ti, and Nb waveforms), with a simultaneous increase in the intensity of the signal from the elements of the magnetic component (maxima of the Fe, Ni, and Zn waveforms). The opposite tendency is observed in the area of the matrix of the ferroelectric composite. The EPMA method of visualizing the surface of the composite microstructure of the sample creates an image (map) of the distribution of individual elements in an illustrative manner. An area with a magnetic grain surrounded by a ferroelectric composite matrix was selected for the analysis (Figure 5d). The mappings expose areas rich in magnetic components (Fe, Ni, and Zn) and rich in ferroelectric components (Pb, Ba, Zr, Ti, and Nb). The presented studies complement each other, showing the distribution of the magnetic and ferroelectric phases in a multiferroic composite sample. The BSE technique, as well as point and linear EDS analyses, show the most significant compliance. On the other hand, EPMA maps can provide an approximate visualization of the microstructure of the ferroelectric-ferromagnetic composite samples.

**Figure 6:**
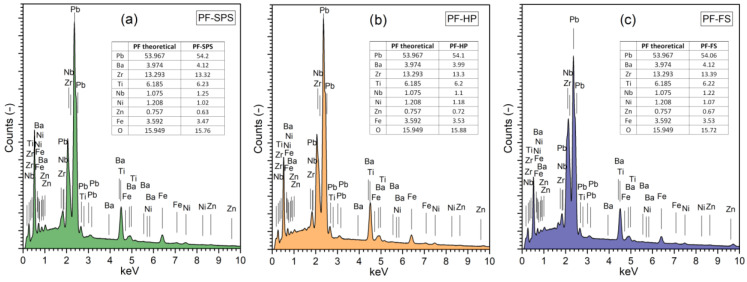
The EDS surface analysis for the PF composite samples: PF-SPS (**a**), PF-HP (**b**), and PF-FS (**c**).

The EDS surface tests (Figure 6) on the larger measuring area of the sample (at low ×1500 magnification) were performed to verify the correctness of obtaining the assumed composition of multiferroic composites. The theoretical percentages of elements for individual elements were compiled based on the stoichiometric calculations of chemical compositions by the chemical reaction of the obtaining thereof. The results of the EDS tests (tables inside Figure 6) are the average values of five randomly selected measurement areas. The EDS analysis showed a slight excess of the ferroelectric component (Pb, Ba, Zr, Ti, and Nb) and a slight deficiency of the magnetic component (Ni, Zn, and Fe). Both in the case of the ferroelectric and the magnetic components, these deviations are within the permissible error. At the same time, the EDS analysis confirmed the absence of foreign elements. The most stable chemical composition (closest to the theoretical one) shows a composite sample sintered by hot pressing method (PF-HP).

The density of multiferroic composite samples determined by the Archimedes method is 6.26 × 10^3^ kg/m^3^ for PP-SPS, 7.12 × 10^3^ kg/m^3^ for PF-HP, and 6.72 × 10^3^ kg/m^3^ for PF-FS. Considering the sinterability during the technological process, the correct compaction of the material, and the highest density, the HP hot sintering method is the most suitable technology for multiferroic composites. Appropriate densification of the multiferroic composite sample ensures the correct microstructure is obtained, thanks to which a material with optimal and stable physical properties and high resistance to electrical breakdown is obtained.

### 3.4. Ferroelectric Properties and Electric Conductivity of Composite Samples 

**Figure 7:**
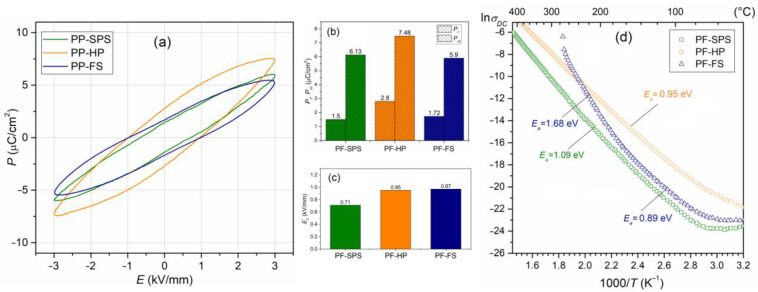
Hysteresis *P*-*E* loops (5 Hz, *RT*) (**a**), values of the residual and maximum polarizations (**b**), coercive field (**c**), and the ln*σ*_DC_(1000/*T*) dependences (**d**) for the PP-F ceramic composites.

Figure 7a shows *P-E* hysteresis loops for the PF composite samples made at *RT* (5 Hz). The presence of ferrite in the PF multiferroic composites reduces the residual polarization, the coercive field, and the correct saturation of the hysteresis loop. The values of the *P_r_* residual polarization are 1.50, 2.80, and 1.72 µC/cm^2^, for PF-SPS, PF-HP, and PF-FS samples, respectively (Figure 7b), while the *E_c_* coercive field is 0.71, 0.95, and 0.97 kV/mm for PF-SPS, PF-HP, and PF-FS samples, respectively (Figure 7c). The composite sample, sintered by hot pressing, retains the most ferroelectric properties (PF-HP), and shows the highest resistance to electric breakdown. Table 1 summarizes the values of the residual polarization, maximum polarization, and the coercive field for composite materials.

At *RT*, the composite samples have correspondingly high values of the *ρ_DC_* resistivity, i.e., 1.35 × 10^9^ Ωm (for PF-SPS), 7.86 × 10^9^ Ωm (for PF-HP), and 2.39 × 10^9^ Ωm (for PF-FS). Above 50 °C, with increased temperature, there is a systematic increase in DC electrical conductivity (Figure 7d), which is related to the increased drift mobility of thermally activated charge carriers [74]. In the case of PF-SPS and PF-HP composite samples, the observed increase shows a stable trend, while in the PF-FS sample above 200 °C, the increase in conductivity is much more rapid. The temperature dependence of DC electrical conductivity obeys the Arrhenius law (1) very well. The activation energies *E_a_* of the multiferroic composite samples were calculated according to the linear slope of the ln*σ*_DC_(1000/*T*) curves and the Arrhenius Equation (1).

(1) $$\sigma_{\mathrm{DC}}=\sigma_{0}\exp\left(\frac{E_{a}}{k_{B}T}\right),\;$$

where: *σ*_0_—pre-exponential factor, *k_B_*—Boltzmann’s constant, *T*—absolute temperature, *E_a_*—the activation energy [75]. 

The calculated activation energy for PF multiferroic composites shows lower values than the P ceramics (1.45 eV), i.e., 1.09 and 0.95 eV for PF-SPS, and PF-HP, respectively. In the case of the PF-FS sample, the activation energy was calculated for two areas of the curve, namely 0.89 eV (at a lower temperature) and 1.68 eV (at a higher temperature). Generally, in the case of perovskite-type materials, DC conductivity is mainly associated with oxygen and lead vacancies, defect dipolar effects, etc. [76,77], whereas, in the case of ferrite materials, it is connected with the hopping mechanism [78,79]; i.e., the hopping of charge carriers between the iron ions available in different valence states [80]. Volatile components can easily create the oxygen vacancies (V_O_) at high temperatures during sintering, leading to the creation of singly/doubly ionized oxygen vacancies $V_{O}^{\prime }/V_{O}^{\prime \prime }$, an ionization of which creates conducting electrons [75]. The calculation *E_a_* shows that the thermal excitation of carriers dictates the electrical conduction from the second ionization of oxygen vacancies. This suggests that the oxygen vacancies migration dominates the electrical conductivity, changing from a single to a doubly ionized mechanism around the ferroelectric phase transition [75]. The presence of ferrite in the multiferroic composite samples makes the materials more conductive (higher hopping rate of charge carriers), especially for the FS method. 

### 3.5. Dielectric Properties of Composite Samples 

#### 3.5.1. Temperature Dependence of Dielectric Properties

**Figure 8:**
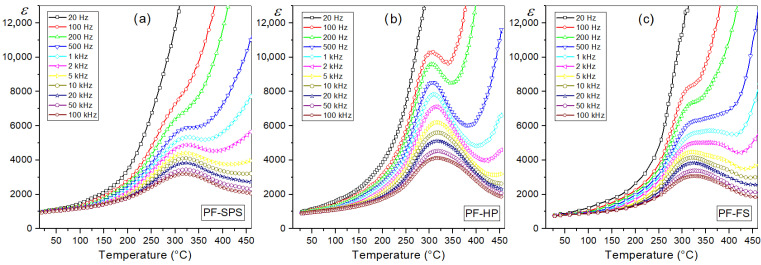
Temperature dependencies of permittivity for the PF multiferroic composites (heating): PF-SPS (**a**), PF-HP (**b**), and PF-FS (**c**).

Ferroelectric P ceramics have high permittivity and low values of dielectric loss factor (tan*δ*), and the ferroelectric phase transformation takes place in a narrow temperature range (Figure 1c,d). The connection to form a multiferroic composite, two materials, i.e., ferroelectric and ferrite materials, reduces the permittivity values; however, constantly, they remain at a sufficiently high level (Table 1). For *RT ε* it is 1000 for PF-SPS, 910 for PF-HP, and 750 for PF-FS, while at *T_m_* it is 5340, 7850, and 5720, for PF-SPS, PF-HP, and PF-FS, respectively. Furthermore, the width of the temperature interval in which the phase transition takes place is significantly widened, and for lower frequencies of the measurement field, the *ε*(*T*) waveforms in the area of the phase transition are blurred (Figure 8). The PF-HP sample is characterized by a lower degree of phase transition blurring, as it retains the highest permittivity values from the analyzed series (Figure 8b). The obtained values of the permittivity of composite materials are higher than those obtained in the works [81], and the phase transition blurring is much smaller.

**Figure 9:**
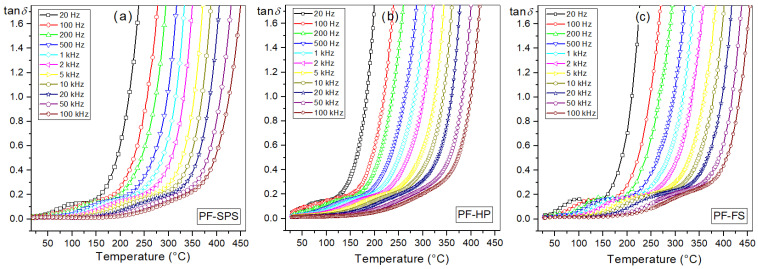
Temperature dependencies of dielectric loss factor tan*δ* for the PF multiferroic composites (heating): PF-SPS (**a**), PF-HP (**b**), and PF-FS (**c**).

Temperature dielectric measurements have also shown that the sintering method used in the PF technological process of multiferroic composites does not cause significant changes in the value of the dielectric loss factor (Figure 9). The dielectric loss factor (tan*δ*) is related to the dielectric relaxation process and is given as the ratio of the imaginary part *ε*″ and the real part *ε*′ (tan*δ* = *ε*″/*ε*′). The *ε*″ (dielectric loss) represents the energy loss and occurs when the polarization shifts behind the applied electric field caused by the grain boundaries [82]. In dielectric materials, the dielectric loss originates from the following factors: space charge migration (interfacial polarization contribution), DC direct current conduction, and the movement of the molecular dipoles (dipole loss) [78]. At *RT*, the tan*δ* values of multiferroic composite samples (Table 1) are in the range of 0.013–0.016 (compared with P ceramics, tan*δ* = 0.013 at *RT*). In contrast, the increase in dielectric loss of composite samples at higher temperatures is more significant (see Figure 1d). The dielectric loss factor values obtained by the three methods (FS, HP and SPS) are much lower than the tan*δ* values for composite materials synthesized by a powder-in-sol precursor hybrid processing route [81].

#### 3.5.2. Frequency Dependence of Dielectric Properties

**Figure 10:**
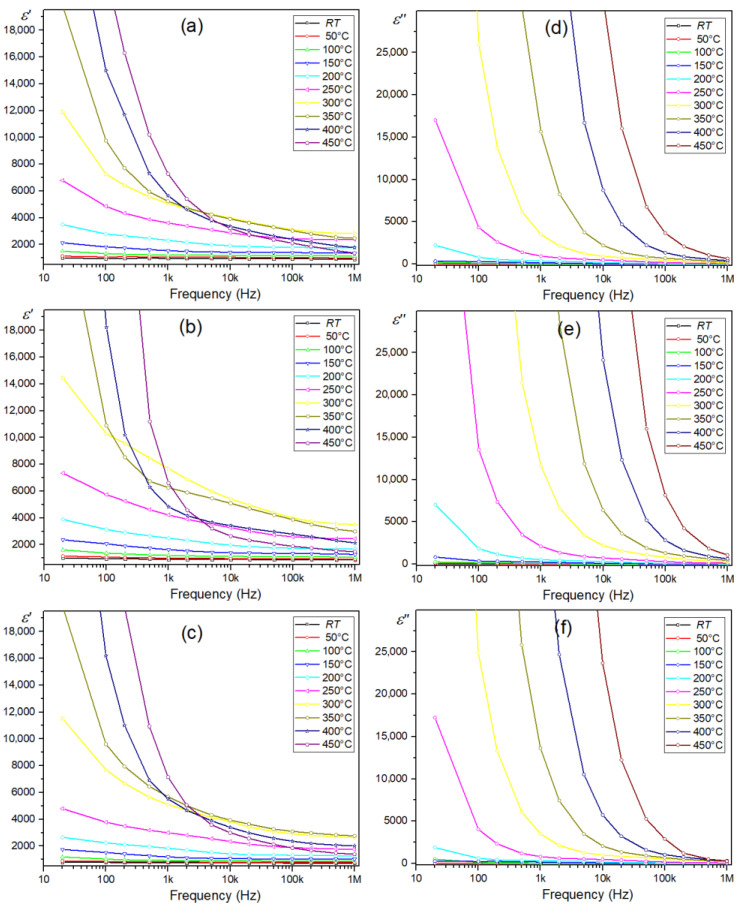
Frequency dependence of real part of permittivity *ε*′ (**a**–**c**) and dielectric loss *ε*″ (**d**–**f**) for the PF multiferroic composites: PF-SPS (**a**,**d**), PF-HP (**b**,**e**), and PF-FS (**c**,**f**).

In the frequency dependence graphs of the real part of permittivity (*ε*′) for multiferroic composite materials (Figure 10a–c), the *ε*′ decreased fast with the increase in frequency. With a further increase in frequency, *ε*′ remains nearly constant. The plots also show that *ε*′ increased with temperature growth (for a specific frequency). The phenomenon of the dielectric dispersion can be explained by the dominance of the grain boundaries’ effect (rather than by the grains) and is attributed to the Maxwell-Wagner type of interfacial polarization based on Koop’s phenomenological theory [83]. According to this model, the microstructure of polycrystalline ceramics consists of semiconducting grains separated by an insulating layer of grain boundaries. Defects arising in the ceramic sample during the technological process, i.e., oxygen vacancies, create space charges at the interface between the sample and the electrode’s space-charge polarization, whose polarization responds to the applied electric field [76]. These charges have enough time at low frequencies to move longer distances in the sample, creating larger electronic polarization (a high dielectric constant value). As the frequency increases, the charge carriers cannot follow the applied external field, and polarizability decreases (value of dielectric constant decreases) [75]. The frequency dependence of the dielectric loss (*ε*″) at different temperatures is shown in Figure 10e–f. The *ε*″(*f*) curves show similar behavior to *ε*′(*f*). The high value of *ε*″ at a low frequency can be attributed to the high resistivity of grain boundaries. All analyzed multiferroic composite samples show similar behavior *ε*″(*f*) curves.

### 3.6. Magnetic Properties of Composite Samples 

**Figure 11:**
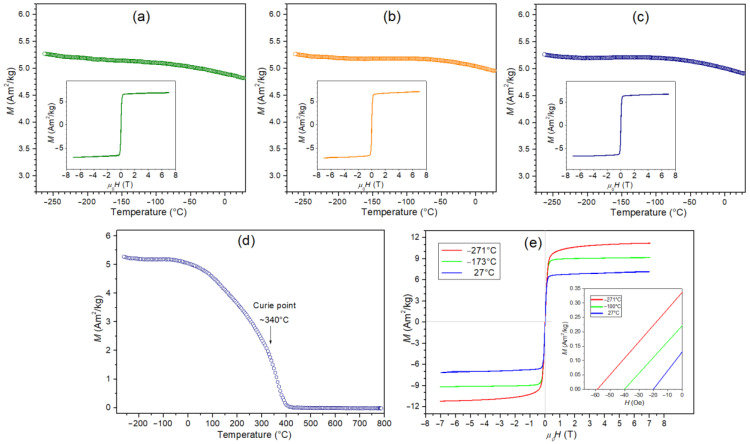
Temperature dependencies of *M* magnetization for PF composite samples: PF-SPS (**a**), PF-HP (**b**), and PF-FS (**c**), inset—magnetic hysteresis loops tested at *RT*; temperature dependencies of *M* magnetization (**d**) and magnetic hysteresis loops for the PF-FS composite sample (**e**), inset—coercivity in a narrower temperature range.

Figure 11a–c shows the temperature dependencies of *M* magnetization in an external magnetic field of 0.1 T (in the temperature range from –268 to 30 °C) for a series of ceramic composite samples. For the composite sample, the highest magnetization values occur at very low temperatures (–268 °C) and then slightly decrease with increasing temperature. The magnetization values in the measured temperature range are from 5.3 to 4.85 Am^2^/kg (Figure 11, Table 1). Temperature measurements of magnetization show dependence typical of ferroelectric-ferromagnetic composite materials, i.e., a strong signal from the ferrimagnetic phase and a weak signal from the paramagnetic phase [42]. Because multiferroic composites have the same amount of ferrite, all examined samples reveal the same magnetic characteristics. The type of synthesis method used does not firmly affect the magnetic properties of the multiferroic composite, as does the type of ferroelectric phase [69]. Magnetic properties of the nickel-zinc ferrite depend from many factors, i.e., on the exchange interactions between octahedral and tetrahedral sub-lattices, magneto-crystalline anisotropy, spin-canted effect, super-exchange interaction, and dipolar interactions between the moments [84]. Therefore, a more detailed study of the magnetic properties was performed on an exemplary sample (PF-FS). Figure 11d shows the *M*(*T*) test measured over a temperature range from −268 to 780 °C. A characteristic well-defined at the Curie point (ferro/ferri to paramagnetic phase transition) can be observed on the curve. In this case, the Curie temperature, determined as an inflection point of d*M*/d*t* curve, is ~340 °C. Figure 11e shows a set of magnetic hysteresis loops measured at different temperatures for the PF-FS sample. The shapes of the hysteresis are typical for soft magnetic materials with saturation magnetization *M*_s_ equal 10.6, 8.9, and 6.75 Am^2^/kg, and coercivity 59, 40, and 20 Oe, for −268, −173, and 27 °C, respectively. A similar shape of the magnetic loop was presented in [85] for other mutiferroic composites but with a much lower magnetization value for the composition with 90/10 (ferroelectric/ferrimagnetic) content compared to the multiferroic composites in this study.

## 4. Conclusions

In this study, multiferroic composite samples were successfully obtained using three different sintering methods, i.e., spark plasma sintering (SPS), hot pressing (HP), and free sintering (FS). The multiferroic composite materials consisted of 90% ferroelectric material (Pb_0_._90_Ba_0_._10_(Zr_0_._53_Ti_0_._47_)O_3_ + 2%at.Nb_2_O_5_) and 10% ferrite (Ni_0_._64_Zn_0_._36_Fe_2_O_4_). In the case of ferroelectric materials, the XRD tests detected peaks originating from tetragonal and rhombohedral phases, while ferrite material detected peaks originating from a cubic spinel crystal structure. The research has shown that the multiferroic composite materials obtained by three methods exhibit good ferroelectric and magnetic properties. The multiferroic composite materials acquire magnetic properties while maintaining good dielectric properties; however, the ferroelectric properties deteriorate. When comparing the test results of the three sintering methods, it can be concluded that a sample sintered with the HP hot pressing method shows the best set of physical parameters. The PF-HP composite sample has high permittivity 910 (at *RT*) and 7850 (at the *T_m_*), high residual polarization 2.80 µC/cm^2^, a coercive field of 0.95 kV/mm, and magnetization 5.3 and 4.95 Am^2^/kg at −268 °C and *RT*, respectively. The optimal technological process conditions are ensured by the HP method, improving the sinterability of the ceramic sinter, which obtains high density and proper material compaction. The other two sintering methods (SPS and FS) show some disadvantages that affect the final properties of composite materials. In the case of the SPS method, the sintering conditions do not allow for simultaneous homogeneous growth of the ferroelectric and magnetic component grains, increasing the formation of internal pores. Similarly, in the FS method, high temperatures favor excessive grain growth and a rise in grain size heterogeneity. In obtaining the optimal final parameters of multiferroic composites and maintaining their high stability, hot pressing is the most effective of the presented sintering methods. Appropriate densification of the multiferroic composite sample ensures that the correct microstructure is obtained. Thanks to the aforementioned (apart from optimal physical properties), multiferroic composite materials with high resistance to electrical breakdown are obtained. The properties of multiferroic composites predispose this group of materials to be used in microelectronics and micromechatronics, e.g., as elements for the construction of various types of sensors, phase modulators, magnetoelectric transducers, and piezoelectric-magnetostrictive accelerometers.

## Figures and Tables

**Table 1:** Parameters of the PF multiferroic composites.

Parameter	PF-SPS	PF-HP	PF-FS
*ρ* (kg/m^3^) ^1^	6.26 × 10^3^	7.12 × 10^3^	6.72 × 10^3^
*r_a_* (μm)	1.52	1.60	2.46
*ρ_DC_* (Ωm) ^1^	1.35 × 10^9^	7.86 × 10^9^	2.39 × 10^9^
*M* (Am^2^/kg) ^2^	5.27	5.30	5.26
*M* (Am^2^/kg) ^1^	4.85	4.95	4.97
*T_m_* (°C) ^3^	327	309	350
*ε* ^1, 3^	1000	910	750
*ε_m_* ^3^	5340	7850	5720
tan*δ* ^1, 3^	0.013	0.016	0.014
*E_a_* (eV)	1.09	0.95	0.89
*P_r_* (µC/cm^2^) ^1, 4^	1.50	2.80	1.72
*P_m_ (*µC/cm^2^) ^1, 4^	6.13	7.48	5.90
*E_c_* (kV/mm) ^1, 4^	0.71	0.95	0.97

^1^ at *RT*, ^2^ at –268 °C, ^3^ for 1 kHz, ^4^ for *E* = 3 kV/mm.

## Data Availability

Data is contained within the article.
